# 67 Methamphetamine-Positive Burn Patients: Not Just an Urban Problem

**DOI:** 10.1093/jbcr/irae036.059

**Published:** 2024-04-17

**Authors:** Allison Proffitt, John H Sojka, Jason Heard, Soman Sen, Tina L Palmieri, David G Greenhalgh, Kathleen S Romanowski

**Affiliations:** UC Davis Health, Sacramento, California; Arizona Burn Center, Phoenix, AZ; University of California - Davis Hosp, Sacramento, CA; UC Davis, Sacramento, CA; UC Davis, Shriners Children's Northern California, Sacramento, CA; Shriners Hospital Northern California, Sacramento, CA; UC Davis and Shriners Children's Northern California, Sacramento, CA; UC Davis Health, Sacramento, California; Arizona Burn Center, Phoenix, AZ; University of California - Davis Hosp, Sacramento, CA; UC Davis, Sacramento, CA; UC Davis, Shriners Children's Northern California, Sacramento, CA; Shriners Hospital Northern California, Sacramento, CA; UC Davis and Shriners Children's Northern California, Sacramento, CA; UC Davis Health, Sacramento, California; Arizona Burn Center, Phoenix, AZ; University of California - Davis Hosp, Sacramento, CA; UC Davis, Sacramento, CA; UC Davis, Shriners Children's Northern California, Sacramento, CA; Shriners Hospital Northern California, Sacramento, CA; UC Davis and Shriners Children's Northern California, Sacramento, CA; UC Davis Health, Sacramento, California; Arizona Burn Center, Phoenix, AZ; University of California - Davis Hosp, Sacramento, CA; UC Davis, Sacramento, CA; UC Davis, Shriners Children's Northern California, Sacramento, CA; Shriners Hospital Northern California, Sacramento, CA; UC Davis and Shriners Children's Northern California, Sacramento, CA; UC Davis Health, Sacramento, California; Arizona Burn Center, Phoenix, AZ; University of California - Davis Hosp, Sacramento, CA; UC Davis, Sacramento, CA; UC Davis, Shriners Children's Northern California, Sacramento, CA; Shriners Hospital Northern California, Sacramento, CA; UC Davis and Shriners Children's Northern California, Sacramento, CA; UC Davis Health, Sacramento, California; Arizona Burn Center, Phoenix, AZ; University of California - Davis Hosp, Sacramento, CA; UC Davis, Sacramento, CA; UC Davis, Shriners Children's Northern California, Sacramento, CA; Shriners Hospital Northern California, Sacramento, CA; UC Davis and Shriners Children's Northern California, Sacramento, CA; UC Davis Health, Sacramento, California; Arizona Burn Center, Phoenix, AZ; University of California - Davis Hosp, Sacramento, CA; UC Davis, Sacramento, CA; UC Davis, Shriners Children's Northern California, Sacramento, CA; Shriners Hospital Northern California, Sacramento, CA; UC Davis and Shriners Children's Northern California, Sacramento, CA

## Abstract

**Introduction:**

Our previous research on burn patients who test positive for methamphetamines (MA) demonstrated that these patients come from areas with higher rates of poverty and lower median incomes. Our current study sought to replicate our previous results as well as further examine the communities from which our patients come with respect to their Social Vulnerability Index and Rural vs. Urban background. We hypothesized that MA-positive patients at our regional burn center would come from more highly disadvantaged urban areas.

**Methods:**

Following IRB approval, a retrospective chart review was conducted using electronic medical records for burn patients admitted from January 2015 to December 2019. MA-positive patients were matched with MA-negative patients based on age and total body surface area (TBSA) burn. Data on Poverty levels, Median Income, Social Vulnerability Index (SVI), and Rural-Urban Commuting Areas were determined using patients’ home zip codes. Analysis was conducted with SAS statistical software, version 9.4 (SAS Institute, Cary, NC, USA) using Student t-test, Chi-square, Fisher Exact, and Wilcoxon 2-sample tests. Results are presented as median (interquartile range).

**Results:**

We matched 107 MA-positive patients with 107 MA-negative controls based on TBSA burn and age. The median age of all patients was 49 (20) years old, and the median burn size was 22.3% (32.5). MA-positive patients experienced higher rates of Poverty (21.4% (13.1) vs 12.8% (12.4); p< 0.0001), increased SVI (0.74 (0.42) vs 0.55 (0.47); p=0.0002) and lower Median Incomes ($49,520 (23,447) vs $59,693 (32,075); p< 0.0001) than their MA-negative counterparts. There was no significant difference between Urban and Rural backgrounds for MA-positive and MA-negative patients (p=0.6). Most MA-positive patients came from Urban locations (16.82% Rural vs. 83.18% Urban), as did most MA-negative patients (19.63% Rural vs. 80.37% Urban). Meth-positive patients were more likely to sustain burns in an incident of suspected assault or abuse (7 vs. 2 patients) and less likely to be burned in a work-related incident (1 vs. 13 patients). Despite these differences in socioeconomic status, MA-positive patients had no significant increase in Length of Stay, Hospital Charges, or Mortality.

**Conclusions:**

MA-positive patients come from both rural and urban areas, areas with increased poverty, lower median incomes, and increased social vulnerability when compared to MA-negative patients. Despite this MA use, by itself, does not appear to change outcomes following burn injury.

**Applicability of Research to Practice:**

Patients with MA use come from both rural and urban areas and are often more socially disadvantaged than patients who do not use MA. These factors should be considered in inpatient care, disposition, and follow-up plans.

